# Congenital Diaphragmatic Hernia Presenting with Tension Pneumothorax in a 3-Year-Old Boy

**DOI:** 10.1055/s-0038-1667357

**Published:** 2018-08-22

**Authors:** Maren Friederike Balks, Jan-Hendrik Gosemann, Ina Sorge, Martin Lacher, Franz Wolfgang Hirsch

**Affiliations:** 1 Departement of Pediatric Surgery, Universitatsklinikum Leipzig, Leipzig, Sachsen, Germany; 2 Departement of Pediatric Radiology, Universitatsklinikum Leipzig, Leipzig, Sachsen, Germany

**Keywords:** tension, pneumothorax, congenital, diaphragmatic, hernia

## Abstract

We report the case of a 3-year-old boy who presented with an upper respiratory tract infection and severe dyspnea. A chest X-ray revealed a left-sided tension pneumothorax with mediastinal shift and suspected enterothorax. After thoracic computed tomography (CT) scan, a chest tube was inserted, which drained fluid which had the same consistency and color as the one derived from the nasogastric (NG) tube. The boy underwent diagnostic laparoscopy for suspected bowel perforation, which confirmed a left-sided Bochdalek hernia with herniation of the viscera into the chest. After repositioning of the herniated organs into the abdomen, a gastric perforation was identified and repaired. This case demonstrates that the cause of a tension pneumothorax in an infant may be a rare combination of congenital diaphragmatic hernia (CDH) and perforation of a visceral hollow organ.

## Introduction

Congenital diaphragmatic hernia (CDH) is a birth defect of the diaphragm caused by an insufficient closure of the pleuroperitoneal canal during fetal development. 1 About 80% of these malformations are located on the left posterolateral side and are therefore called Bochdalek hernias. 2 Most patients with CDH are diagnosed prenatally or immediately after birth. 3

However, children with small defects may be asymptomatic postnatally and present later in life, when an increased intra-abdominal pressure facilitates herniation of organs into the chest. 4 5 6 Some cases of CDH develop gastrointestinal symptoms due to gastric ischemia or injury or volvulus. 3 7 We report on a child with previously unknown Bochdalek hernia and life-threatening presentation due to tension pneumothorax.

## Case Report

**Fig. 1:**
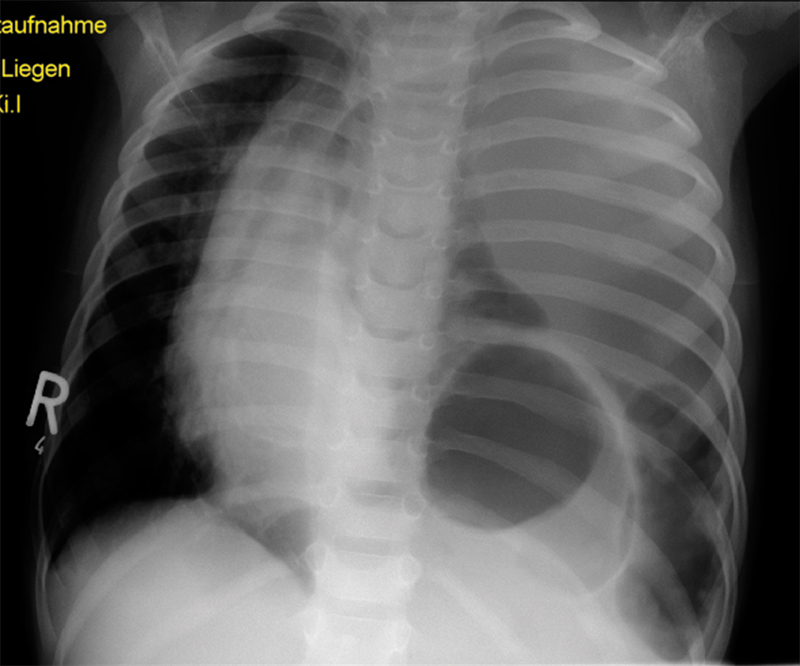
Chest X-ray at presentation (this image is provided by courtesy of Pediatric Clinic, Fachkrankenhaus Hubertusburg, Wermsdorf, Sachsen, Germany).

A 3-year-old boy presented to an outside hospital with a 5-day history of progressing respiratory distress and retching. The initial chest X-ray showed a left-sided tension pneumothorax with mediastinal shift and the suspicion of bowel loops in the left lower hemithorax ( Fig. 1 ). Therefore, the patient was transferred to our institution.

**Fig. 2:**
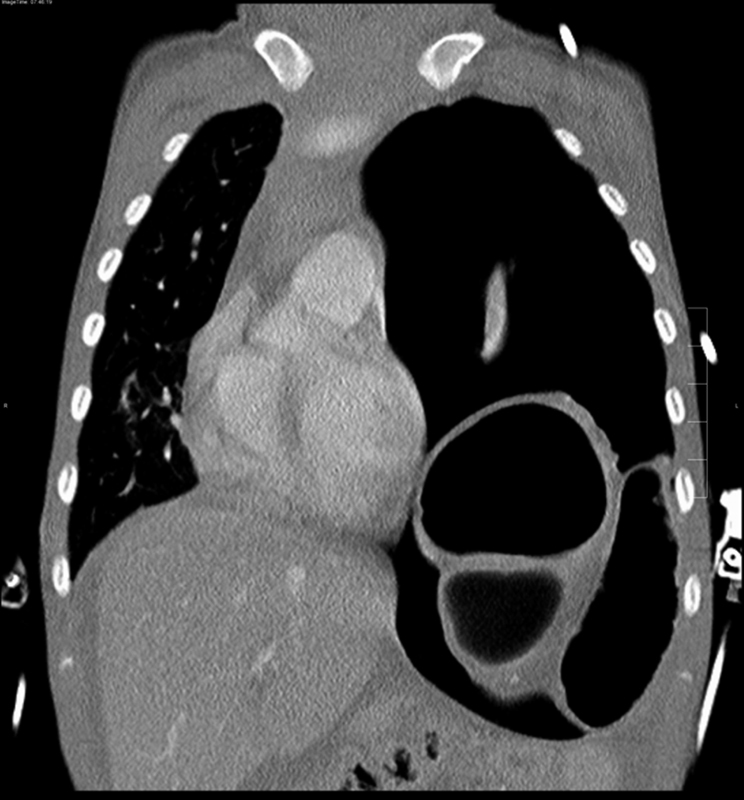
Thoracic CT scan on admission: left-sided tension pneumothorax with mediastinal shift. No further signs of a traumatic etiology.

**Fig. 3:**
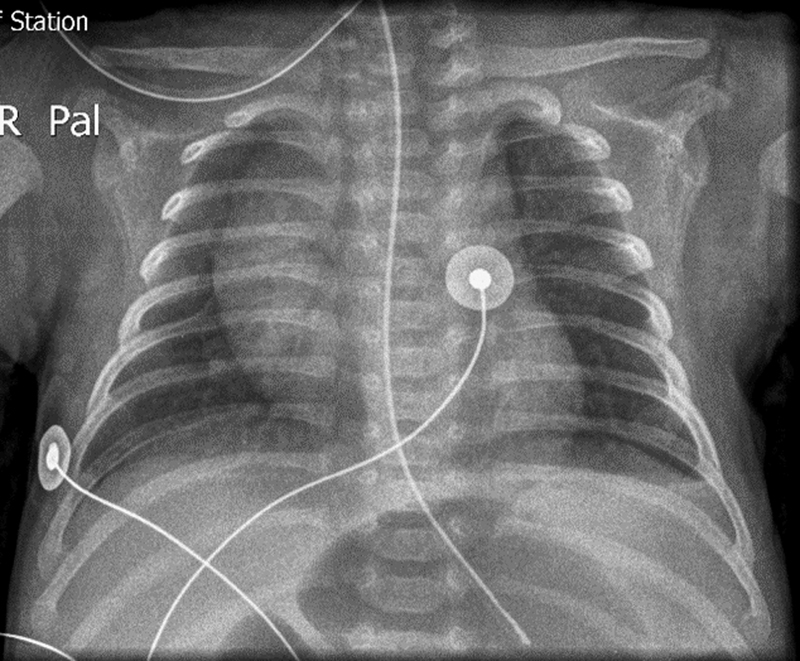
Chest X-ray at 1-year of age with no signs of diaphragmatic hernia.

**Fig. 4:**
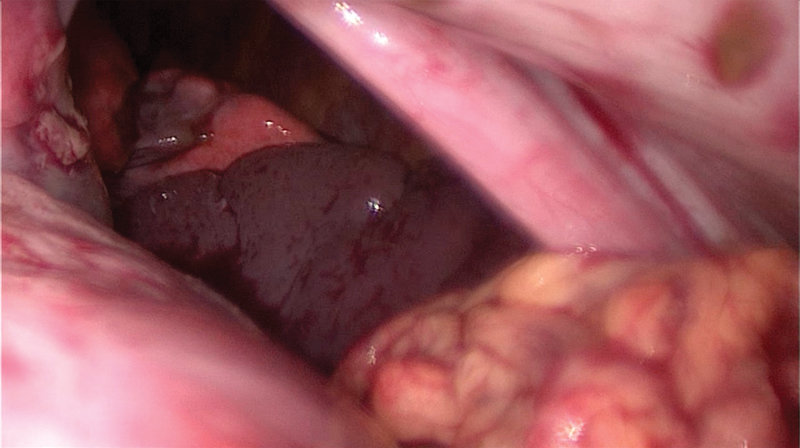
Diagnostic laparoscopy: herniation of stomach, spleen and bowel into the chest.

**Fig. 5:**
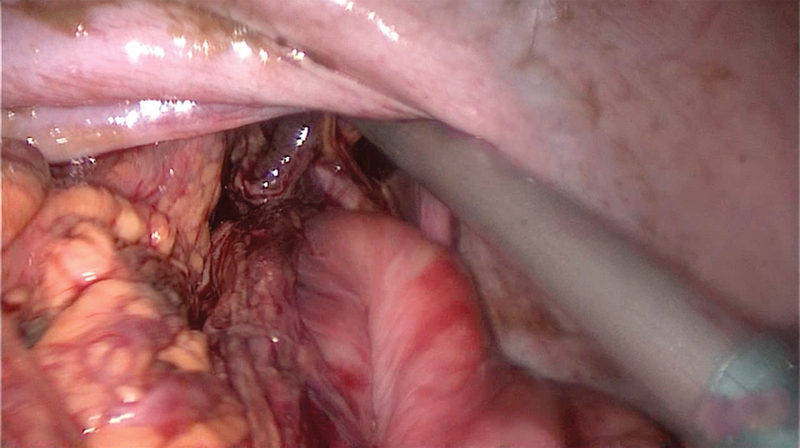
Gastric perforation after repositioning of herniated organs into the abdomen.

On admission, he showed severe dyspnea, a temperature of 39.5°C and tachycardia of 200/min. After immediate endotracheal intubation, a thoracic computed tomography (CT) scan was performed, which confirmed a left-sided enterothorax with mediastinal shift ( Fig. 2 ). A left-sided chest tube was inserted, which drained a fluid that was initially considered to be old blood. Due to the sudden onset of symptoms and a normal chest X-ray which was available from the age of 1 year ( Fig. 3 ), a diaphragmatic rupture was considered as a differential diagnosis. The boy was therefore taken to the operation room (OR) immediately. On diagnostic laparoscopy, a left-sided Bochdalek hernia was detected with herniation of the small intestine, spleen, and stomach into the chest ( Fig. 4 ). Bile-stained fluid was found in the thorax and abdomen. After repositioning of the herniated organs into the abdomen, a gastric perforation at the lesser curvature was detected ( Fig. 5 ), explaining the pneumothorax. The surgeon felt that the gastric perforation could not be closed safely laparoscopically; therefore, a conversion to laparotomy was performed with closure of the gastric perforation and repair of the CDH with interrupted stitches. After extubation on the fourth postoperative day, a retrovesical abscess was drained 30 days after the surgery. Due to a gastroparesis, the boy showed a prolonged recovery and was finally discharged after 4 to 5 weeks in good condition. After a follow-up of 2 years, the boy is asymptomatic and is doing well.

## Discussion

Intestinal symptoms of late presenting CDH can be nonspecific and may include abdominal pain or respiratory symptoms. 3 8 On radiologic imaging, heart murmurs and dilated bowel loops or intrathoracic cysts 3 are not found infrequently. We present the second case of a CDH with tension pneumothorax due to gastric perforation. CDH in association with gastric morbidity has been described in children of different age groups. In a systemic review on 362 children with late-presenting CDH, 46% of these were <1 year old, 32% aged 1 to 5 years, and 22% over 5 years old. 6 Therefore, the patient presented here is in the middle spectrum of these age groups. Gastric complications of CDH include gastric volvulus 7 and an incarceration of the stomach with or without perforation. 4 Baglai et al reported on gastric volvulus being the leading cause of gastric morbidity in late-presenting CDH in 45% followed by gastric incarceration with (35%) and without (20%) perforation. 6 In our case, we speculate that gastric ischemia with perforation in the absence of gastric volvulus was most likely the pathogenesis. However, we cannot rule out a combination of a previous volvulus and subsequent gastric perforation.

Unusual in our case was the clinical presentation with tension pneumothorax, which has been described before; Ozkan et al 5 reported a 5-year-old girl in whom the initial chest X-ray showed a thoracic herniation of the stomach, which was misinterpreted as a lung cyst at the left lower lobe. Two days after discharge, she was readmitted for a left-sided tension pneumothorax. This complication is extremely rare in CDH. In hindsight, as the chest tube in our patient drained the same fluid as did the nasogastric (NG) tube, we could have already thought of a hollow visceral perforation preoperatively. As the second imaging modality, a thoracic CT scan was performed as rupture of the diaphragm was considered a possible differential diagnosis. However, it can be discussed whether the CT scan was necessary, since it did not add additional information to the findings revealed by a conventional X-ray.

CDH may be asymptomatic at birth and may present at a later age. The herniation of the viscera into the chest leads to respiratory symptoms. In case of both, enterothorax and tension pneumothorax, a gastric or bowel perforation in combination with CDH must be taken into consideration.

## Notes

New Insights and the Importance for a Pediatric Surgeon CDH may be asymptomatic at birth and may present at a later age. The herniation of the viscera into the chest leads to respiratory symptoms. In case of both, enterothorax and tension pneumothorax, a gastric or bowel perforation in combination with CDH must be taken into consideration.
